# Rate Adaption for Secure HARQ-CC System with Multiple Eavesdroppers

**DOI:** 10.3390/e22040403

**Published:** 2020-03-31

**Authors:** Yue Wu, Shishu Yin, Jian Zhou, Pei Yang, Hongwen Yang

**Affiliations:** 1 Department of Electronic and Information Engineering, Anhui University of Finance and Economics, Bengbu 233030, China; yin_shishu@163.com (S.Y.); ac_zj_course@163.com (J.Z.); 2 School of Information and Communication Engineering, Beijing University of Posts and Telecommunications, Beijing 100876, China; yp@bupt.edu.cn (P.Y.); yanghong@bupt.edu.cn (H.Y.)

**Keywords:** physical layer security (PLS), hybrid automatic repeat request (HARQ), chase combining (CC), secrecy outage probability (SOP), effective secrecy throughput (EST)

## Abstract

In this paper, we studied the secure transmission of a hybrid automatic repeat request with chase combining (HARQ-CC) system, under the existence of multiple eavesdroppers and limited latency. First, we analyzed some critical performance metrics, including connection outage probability (COP), secrecy outage probability (SOP) and effective secrecy throughput (EST). Then, to maximize the EST, three optimization problems of rate adaption were discussed: (i) optimizing the code rate with a given secrecy redundancy rate by a parameterized closed-form solution; (ii) optimizing the secrecy redundancy rate with a given code rate by a fixed-point method; (iii) optimizing both code rate and secrecy redundancy rate by an iterative optimization algorithm. We also considered COP and SOP constraints among the problems while corresponding solutions were deduced. Finally, numerical and simulated results verified our conclusions that the approximated SOP matches well with Monte–Carlo simulation for a strict reliable constraint, and that the optimized transmitting rate enhances EST efficiently with multiple eavesdroppers and retransmissions. Moreover, the influence of the number of eavesdroppers on secrecy performance was analyzed. Briefly, secrecy performance inevitably deteriorates with increasing number of eavesdroppers due to raised information leakage.

## 1. Introduction

In modern wireless communication systems, physical layer security (PLS) is regarded as a critical aspect in providing confidential message transmission according to the characteristics of wireless channels. Differing from traditional encryption techniques, PLS can be proved and quantified without the risk of brute-force cracking. Shannon first proposed the notion of information-theoretic secrecy in his groundbreaking work [1]. The more practical framework, named ‘the wiretap channel’, was established by Wyner in terms of a binary symmetric channel (BSC) [2]. Another important contribution of Wyner was the designing of a secrecy coding scheme, in which the secrecy redundancy rate worked to confuse an eavesdropper. As extended versions, the wiretap channel has been considered in broadcast channels by Csiszar [3] and in Gaussian channels by Leung-Yan-Cheong [4].

On the basis of the above work, PLS has contributed to considerable progress, especially in performance optimization and signal processing. Secrecy capacity, which evaluates the effectiveness of secure transmission, has been defined as the maximum rate in each reliable and secure transmission [5]. This metric has been adopted in the security analysis and optimization of 5G mmWave small cell networks [6], co-operative non-orthogonal multiple access with proactive jamming [7], artificial noise (AN)-aided multi-input multi-output (MIMO) Rician channels [8] and so on. As the secrecy capacity may be less than target secrecy redundancy rate, the secrecy outage probability (SOP) has been applied to present a more comprehensive performance evaluation. For instance, [9] discussed a security region with AN based on SOP, and [10] minimized SOP in a D2D-enabled cellular network by access control. When a certain SOP constraint is required, secrecy throughput has widely been considered, especially in optimization problems where the transmission rate or power is adjusted for improved security [11,12]. On the other hand, signal processing-related methods have also been proposed, mainly including beamforming and precoding, AN, co-operative and relay and diversity technologies [13,14,15,16].

Recently, many excellent contributions have focused on the PLS of hybrid automatic repeat request (HARQ) methods capable of typical time diversity features. In HARQ with chase combining (HARQ-CC), the transmitter retransmits erroneous codewords (or their redundant versions) if the legitimate receiver fails to decode them. On the contrary, new transmissions are triggered when either the decoding is successful or the maximum transmission number is reached [17]. Due to the increased comprehensive requirements, Makki et al. [18] proposed a low-latency reliable HARQ protocol using finite blocklength codes. When a passive eavesdropper exists, it has been proven that retransmissions and combinations are capable of enhancing security, due to the diversity gain of the legitimate receiver [19]. In order to efficiently design secure HARQ, Tang et al. [20] discussed SOP, secrecy throughput and their asymptotic properties; Tomasin [21] proposed a multiple-encoding HARQ scheme with statistics channel state information (CSI); Mheich et al. and Treust et al. [22,23] optimized secrecy throughput using multi-level feedback and rate adaption. However, most of them did not consider the influence of secrecy outage on secrecy throughput, which generally led to overestimated performance [24]. Hence, in our previous work, we extended the effective secrecy throughput (EST) of a single transmission [25] into a HARQ-CC system with a passive eavesdropper, and optimized secrecy redundancy rate for improved security [26]. Nevertheless, another common scenario, which includes multiple eavesdroppers, rate adaption and limited latency, has not yet been analyzed.

Inspired by this problem, in this paper, we completed the optimization of both the code rate and secrecy redundancy rate to maximize EST in a general scenario with multiple eavesdroppers. At the same time, different latency requirements were also considered. The major contributions of our work include:

- The closed-form expressions of COP, average transmission number and SOP in the HARQ-CC system with multiple eavesdroppers and different latencies are given. The corresponding approximations were also deduced, while EST was defined considering both reliable outage and secrecy outage.
- With a given secrecy redundancy rate, the optimization problem of code rate to maximize the EST was discussed. This problem was solved with a parameterized closed-form solution, with and without the COP constraint.
- When the code rate is given, the optimization problem of secrecy redundancy rate with EST criteria was also analyzed. We solved this problem by applying a fixed-point method, with and without the SOP constraint.
- The joint optimization problem of the rate pair (i.e., code rate and secrecy redundancy rate), in order to maximize the EST, was discussed. To solve this problem, an iterative optimization algorithm was designed which involves the two methods mentioned above. COP and SOP constraints were also considered.
- Numerical and simulated results confirm our expressions of critical secure performance metrics, as well as the proposed optimization methods, under different cases. We also found that secrecy performance inevitably deteriorates with an increasing number of eavesdroppers, due to more information leakage.

The rest of this paper is organized as follows: The overall system model and assumptions are described in Section 2. Section 3 expresses COP, average transmission number, SOP and its approximation, along with the definition of EST in a HARQ-CC system with multiple eavesdroppers. Section 4 proposes the optimization of code rate, secrecy redundancy rate and both of them, in order to maximize EST under different constraints and the numerical and simulated results are presented in Section 5. Section 6 concludes our work.

Notation: E[·] denotes the expectation operator. The function $\Gamma \left(a,x\right)$ is the upper incomplete gamma function, $\Gamma \left(a\right)$ is the gamma function, $\Gamma_{r}\left(a,x\right)=\Gamma \left(a,x\right)/\Gamma \left(a\right)$ and $\gamma_{r}\left(a,x\right)=1-\Gamma_{r}\left(a,x\right)$ are the regularized upper and lower incomplete gamma functions, respectively. N(a,b) denotes a Gaussian distribution with mean *a* and variance *b*, respectively. $f_{X}\left(x\right)$ and $F_{X}\left(x\right)$ denote the probability density function (PDF) and the cumulative distribution function (CDF) of a random variate *X*, respectively. The function $W_{0}\left(x\right)$ is the principal branch ($W_{0}\left(x\right)>-1$) of Lambert’s *W*-function, defined through the implicit equation $x=W\left(x\right)e^{W\left(x\right)}$ [27].

## 2. System Model of Secure HARQ-CC With Multi-Eavesdroppers

**Figure 1:**
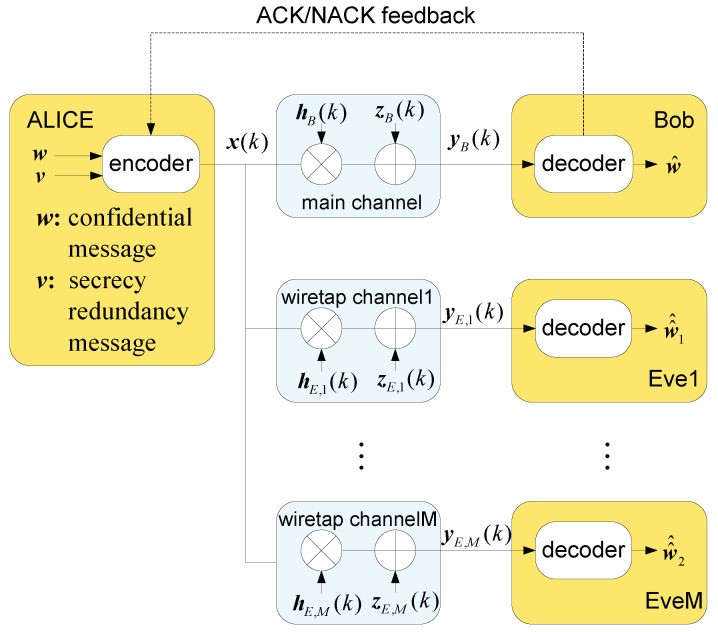
Secure hybrid automatic repeat request (HARQ) with chase combining (HARQ-CC) system model with multiple eavesdroppers.

We considered a secure HARQ-CC transmission system with multiple eavesdroppers, as shown in Figure 1. The transmitter (Alice) sends a confidential message w with a secrecy redundancy message v to the legitimate receiver (Bob) over the main channel, while several passive eavesdroppers (Eve1, …, EveM) intercept the transmission through *M* wiretap channels. We assumed that the main and wiretap channels are independent Rayleigh block-fading channels. Retransmissions are triggered only by Bob, depending on his decoding failure. To avoid unexpected retransmissions, the maximum transmission number (*K*) guarantees limited latency. A major security advantage of this protocol is that the erroneous codewords received by eavesdroppers may not be retransmitted by Alice, unless Bob had the same erroneous ones. Hence, there was much more diversity gain obtained by Bob than Eve1, …, EveM.

Alice encodes the confidential message w and the secrecy redundancy message v into the codeword x(k) using the Wyner secrecy code [2], where *k* is the transmission number (1 ≤ *k* ≤ *K*). The code rate and secrecy redundancy rate are denoted by $R_{B}$ and $R_{E}$, respectively. Thus, the secrecy rate is given by $R_{s}=R_{B}-R_{E}$. Assume that the transmission power is fixed at *P*, and $E\left[{\left|x(k)\right|}^{2}\right]=1$. We denote the fading parameters of the main and wiretap channels by $h_{B}\left(k\right)$ and $h_{E,1}\left(k\right),\ldots ,h_{E,M}\left(k\right)$, respectively, which are independently and identically distributed (i.i.d.) complex Gaussian random variables with zero mean and unit variance. We denote the additive Gaussian white noise by $z_{B}\left(k\right)$ and $z_{E,1}\left(k\right),\ldots ,z_{E,M}\left(k\right)$, respectively. Their means are zero and their variances are, respectively, $\sigma_{B}^{2}$ and $\sigma_{E,1}^{2},\ldots ,\sigma_{E,M}^{2}$. In each slot, the received signals of Bob and Eve1, …, EveM after *k* transmissions are

(1) $$\begin{array}{c} \left\{\begin{array}{cc} y_{B}\left(k\right) & =\sqrt{P}h_{B}\left(k\right)x\left(k\right)+z_{B}\left(k\right)\\ y_{E,m}\left(k\right) & =\sqrt{P}h_{E,m}\left(k\right)x\left(k\right)+z_{E,m}\left(k\right),m=1,\ldots ,M \end{array}.\right. \end{array}$$

For simplicity, we define $S_{B}=P/\sigma_{B}^{2}$ and $S_{E,m}=P/\sigma_{E,m}^{2},\left(m=1,\ldots ,M\right)$ as the average received signal-to-noise ratio (SNR) through the main channel and the wiretap channels, respectively. After *k* transmissions, Bob and Eve1, …, EveM uses the maximal ratio combining (MRC) before decoding. Their combined SNR becomes:

(2) $$\begin{array}{c} \left\{\begin{array}{cc} \gamma_{B}\left(k\right) & =\sum_{i=1}^{k}S_{B}{\left|h_{B}\left(i\right)\right|}^{2}\\ \gamma_{E,m}\left(k\right) & =\sum_{i=1}^{k}S_{E,m}{\left|h_{E,m}\left(i\right)\right|}^{2},m=1,\ldots ,M \end{array}.\right. \end{array}$$

## 3. Security Performance Metrics

Based on the above secure HARQ-CC system model with multiple eavesdroppers, we analyzed some critical security performance metrics, including connection outage probability (COP), average transmission number, secrecy outage probability (SOP) and effective secrecy throughput (EST). Connection outage occurs when the legitimate receiver (Bob) cannot decode the transmitted codewords, and secrecy outage occurs when one or several of the eavesdroppers (Eve1, …, EveM) cannot be confused by secrecy redundancy after the $k^{\mathrm{th}}$ transmission.

We first considered the COP after *k* transmissions, denoted by $P_{e}\left(k\right)$. The COP is defined as the probability that a connection outage occurs; that is, the mutual information after the $k^{\mathrm{th}}$ transmission, $I_{B}\left(k\right)$, is less than the codeword rate $R_{B}$,

(3) $$\begin{array}{c} \begin{array}{cc} P_{e}\left(k\right) & =\mathrm{Pr}\{I_{B}\left(k\right)<R_{B}\}\\ & =\mathrm{Pr}\{\ln\left(1+\sum_{i=1}^{k}{S_{B}\left|h_{B}\left(i\right)\right|}^{2}\right)<R_{B}\}\\ & =\mathrm{Pr}\left\{\sum_{i=1}^{k}{\left|h_{B}\left(i\right)\right|}^{2}<\frac{e^{R_{B}}-1}{S_{B}}\right\} \end{array}. \end{array}$$

We know that the fading parameters are independent zero-mean unit-variance complex Gaussian random variables. Hence, the sum of their modular square is distributed according to the chi-squared distribution. Denote the decoding threshold of the main channel by $\Theta_{B}=\left(e^{R_{B}}-1\right)/S_{B}$, then

(4) $$\begin{array}{c} \begin{array}{cc} P_{e}\left(k\right) & =F_{\chi^{2}}\left[2\Theta_{B},2k\right]\\ & =\gamma_{r}\left(k,\Theta_{B}\right) \end{array}, \end{array}$$

where $F_{\chi^{2}}\left[\cdot \right]$ is the cumulative distribution function (CDF) of a chi-squared random variable.

The average transmission number $\bar{N}$ is determined by the main channel, which is equal to the expectation of the actual transmission number, *N*,

(5) $$\begin{array}{c} \begin{array}{cc} \bar{N} & =E\left[N\right]=1+\sum_{k=1}^{K-1}P_{e}\left(k\right)\\ & =1+\sum_{k=1}^{K-1}\gamma_{r}\left(k,\Theta_{B}\right) \end{array}. \end{array}$$

The SOP of HARQ-CC, denoted by $P_{s}\left(k\right)$, is defined as the probability that a message transmitted by Alice can be decoded successfully by Eve1 or … or EveM after *k* transmissions. As passive receivers, Eve1, …, EveM only receive messages when retransmissions are requested by Bob. When the number of transmissions in the main channel is *N*,

(6) $$\begin{array}{c} \begin{array}{cc} P_{s}\left(k\right) & =\sum_{i=1}^{k}\mathrm{Pr}\left\{N=i\right\}\cdot \mathrm{Pr}\left\{I_{E,1}\left(i\right)>R_{E}⋃I_{E,2}\left(i\right)>R_{E}⋃\ldots ⋃I_{E,M}\left(i\right)>R_{E}\right\}\\ & =\sum_{i=1}^{k}\mathrm{Pr}\left\{N=i\right\}\cdot \left(1-\mathrm{Pr}\left\{I_{E,1}\left(i\right)<R_{E},I_{E,2}\left(i\right)<R_{E},\ldots ,I_{E,M}\left(i\right)<R_{E}\right\}\right) \end{array}, \end{array}$$

where $I_{E,i}$ is the mutual information of the wiretap channel, $\mathrm{Pr}\left\{N=i\right\}$ is the probability that the $i^{\mathrm{th}}$ transmission occurs, and $\mathrm{Pr}\left\{N=i\right\}=P_{e}\left(i-1\right)-P_{e}\left(i\right)$. Assume that *M* wiretap channels are i.i.d. Gaussian block fading channels, $S_{E,m}=S_{E}$, $\mathrm{Pr}\left\{I_{E,m}\left(i\right)>R_{E}\right\}=\mathrm{Pr}\left\{I_{E}\left(i\right)>R_{E}\right\},m=1,\ldots ,M$. Denote the decoding threshold of a wiretap channel by $\Theta_{E}=\left(e^{R_{E}}-1\right)/S_{E}$. Then we define

(7) $$\begin{array}{c} \begin{array}{cc} \phi (i) & =\mathrm{Pr}\left\{I_{E,1}\left(i\right)<R_{E},I_{E,2}\left(i\right)<R_{E},\ldots ,I_{E,M}\left(i\right)<R_{E}\right\}\\ & =\mathrm{Pr}\left\{I_{E,1}\left(i\right)<R_{E}\right\}\mathrm{Pr}\left\{I_{E,2}\left(i\right)<R_{E}\right\}\ldots \mathrm{Pr}\left\{I_{E,M}\left(i\right)<R_{E}\right\}\\ & ={\left(\mathrm{Pr}\left\{I_{E}\left(i\right)<R_{E}\right\}\right)}^{M}\\ & ={\left(\mathrm{Pr}\left\{\log_{2}\left(1+\sum_{j=1}^{i}{S_{E}\left|h_{E,m}\left(i\right)\right|}^{2}\right)<R_{E}\right\}\right)}^{M}\\ & ={\left(\gamma_{r}\left(i,\Theta_{E}\right)\right)}^{M} \end{array}. \end{array}$$

Hence, the SOP after *K* transmissions becomes

(8) $$P_{s}\left(K\right)=\sum_{i=1}^{K}\mathrm{Pr}\left\{N=i\right\}\cdot \left(1-\phi (i)\right).$$

It is well-known that an extremely small COP is the fundamental reliablity requirement in modern systems. We assume that under different latency requirements (i.e., different maximum transmission numbers *K*), a small $P_{e}\left(K\right)$ has to be assured. Thus we have $\sum_{i=1}^{K}\mathrm{Pr}\left\{N=i\right\}=1$, then

(9) $$\begin{array}{c} \begin{array}{cc} P_{s}\left(K\right) & =\sum_{i=1}^{K}\mathrm{Pr}\left\{N=i\right\}-\sum_{i=1}^{K}\mathrm{Pr}\left\{N=i\right\}\cdot \phi \left(i\right)\\ & =1-E[\phi (N)] \end{array}. \end{array}$$

As *N* is an integer, ϕ(N) is also $M^{\mathrm{th}}$ power of the complementary CDF (CCDF) of a Poisson random variable. With a given $R_{E}$, the PDF and CDF are both well-known as a log-concave function of *N*. According to [28], its CCDF is also log-concave. In other words, lnϕ(N) is concave with respect to *N*. Under the above assumption that a low $P_{e}\left(K\right)$ is assured under different *K*, $\sum_{i=1}^{K}\mathrm{Pr}\left\{N=i\right\}=1$. According to Jenson’s inequality, we have

(10) $$\begin{array}{c} \begin{array}{cc} \ln\phi \left(E[N]\right) & \leq E\left[\ln\phi (N)\right]\\ & =\sum_{i=1}^{K}\mathrm{Pr}\left\{N=i\right\}\cdot \ln\phi (N)\\ & =\sum_{i=1}^{K}\ln{\phi (N)}^{\mathrm{Pr}\left\{N=i\right\}}\\ & =\ln\prod_{i=1}^{K}\phi {\left(i\right)}^{\mathrm{Pr}\left\{N=i\right\}}\\ & \overset{\left(a\right)}{\leq }\ln\sum_{i=1}^{K}\mathrm{Pr}\left\{N=i\right\}\cdot \phi \left(i\right)\\ & =\ln E\left[\phi (i)\right]\\ & =\ln\left(1-P_{s}\left(K\right)\right) \end{array}, \end{array}$$

where (*a*) is true, based on the general mean inequality, and $1-P_{s}\left(K\right)$ and $\phi \left(E[N]\right)$ are both positive. Thus,

(11) $$P_{s}\left(K\right)\leq 1-\phi \left(E[N]\right).$$

Substituting Equation (7) into Equation (11), we approximate the SOP of the $K^{\mathrm{th}}$ transmission by its upper bound, as follows:

(12) $$P_{s}\left(K\right)≃1-{\left(\gamma_{r}\left(K,\Theta_{E}\right)\right)}^{M}.$$

For simplicity, we define $P_{e}=P_{e}\left(K\right)$ and $P_{s}=P_{s}\left(K\right)$, which means that the maximum transmission number of COP and SOP has been reached.

Most related works have not considered the influence of SOP in secrecy throughput, but this has been found to be inaccurate [24]. Hence, we define the effective secrecy throughput (EST) of a secure HARQ-CC system as [26]

(13) $$\eta_{s}=\frac{E[R_{s}]}{E[N]}=\frac{\left(R_{B}-R_{E}\right)\cdot \left(1-P_{e}\right)\cdot \left(1-P_{s}\right)}{\bar{N}},$$

where $R_{s}=R_{B}-R_{E}$ indicates the maximum rate of each reliable and secure transmission, $\bar{N}$ is the average transmission number, and the COP and SOP are denoted by $P_{e}$ and $P_{s}$, respectively. Based on the renewal–reward theorem [29,30], $\eta_{s}$ in Equation (13) expresses the average reliable and secure transmission rate of each transmission. As this metric demonstrates the secrecy performance more comprehensively, we adapted the code rate and secrecy redundancy rate to enhance the performance with this criteria in the following section.

## 4. Rate Adaption in Secure HARQ-CC System

In this section, in order to improve the performance of secure transmission when multiple eavesdroppers exist and latency is limited, we optimized the code rate $R_{B}$ and secrecy redundancy rate $R_{E}$ to maximize the EST of HARQ-CC. Three cases were considered: When $R_{E}$ is given, $R_{B}$ is optimized by a parameterized closed-form solution, with and without a COP constraint. When $R_{B}$ is given, $R_{E}$ is optimized by a fix-point method, with and without an SOP constraint. Combining the above methods, we then solved the joint optimization of the rate pair $\left(R_{B},R_{E}\right)$ by an iterative algorithm with and without both COP and SOP constraints.

### 4.1. Optimization of Code Rate

In a secure HARQ-CC system, when the number of eavesdroppers is *M*, the maximum transmission number is *K* and the secrecy redundancy rate is given by ${\tilde{R}}_{E}$, we first considered the problem of how to determine the code rate which maximizes the EST:

(14) $$\begin{array}{c} \begin{array}{cc} \max_{R_{B}} & \;\eta_{s}\\ s.t. & \;0\leq {\tilde{R}}_{E}\leq R_{B} \end{array}, \end{array}$$

where $P_{e}$, $P_{s}$ and $\eta_{s}$ are obtained by Equations (4), (12) and (13), respectively. Now, we extended the parameterized closed-form solution [17] to solve this problem. Several HARQ schemes tell us the standard solution is to solve the equation $d\eta_{s}/dR_{B}=0$ for the (globally) optimal rate point ${\hat{R}}_{B}$. Furthermore, $d^{2}\eta_{s}/dR_{B}^{2}{|}_{{\hat{R}}_{B}}<0$ is required to guarantee a global maximum.

The basic idea of this solution method is to use the substitution $R_{B}=\ln\left(1+S_{B}\Theta_{B}\right)$ for the rate in the numerator of the EST expression. $S_{B}$ only occurs in the numerator once; hence, instead of considering the rate $R_{B}$, we focus on the threshold $\Theta_{B}$ in the optimization.

The EST expression for secure HARQ-CC is first parameterized with respect to $\Theta_{B}$, according to

(15) $$\begin{array}{c} \begin{array}{c} \eta_{s}=\frac{R_{B}-R_{E}}{\tilde{\bar{N}}\left(\Theta_{B}\right)}=\frac{\ln\left(1+S_{B}\Theta_{B}\right)-{\tilde{R}}_{E}}{\tilde{\bar{N}}\left(\Theta_{B}\right)} \end{array}, \end{array}$$

where $\tilde{\bar{N}}\left(\Theta_{B}\right)\overset{▵}{=}\bar{N}\left(\Theta_{B}\right)/\left(1-P_{e}\left(\Theta_{B}\right)\right)\left(1-P_{s}\left(\Theta_{B}\right)\right)$, and $P_{s}\left(\Theta_{B}\right)$ is also function of $\Theta_{B}$, as $S_{E}$, $R_{E}$, *M* and *K* are given. Then, we take the derivative with respect to $\Theta_{B}$,

(16) $$\begin{array}{c} \begin{array}{c} \frac{d\eta_{s}}{d\Theta_{B}}=\frac{1}{{\left(\tilde{\bar{N}}\left(\Theta_{B}\right)\right)}^{2}}\left(\frac{S_{B}}{1+S_{B}\Theta_{B}}\tilde{\bar{N}}\left(\Theta_{B}\right)-{\tilde{\bar{N}}}^{\prime }\left(\Theta_{B}\right)\left(\ln\left(1+S_{B}{\hat{\Theta }}_{B}\right)-{\tilde{R}}_{E}\right)\right) \end{array}. \end{array}$$

Let $d\eta_{s}/d\Theta_{B}{|}_{{\hat{\Theta }}_{B}}=0$, where ${\hat{\Theta }}_{B}$ is the optimal point, and divide both sides by ${\hat{\Theta }}_{B}$. Then, we have

(17) $$\frac{1+S_{B}{\hat{\Theta }}_{B}}{S_{B}{\hat{\Theta }}_{B}}\left(\ln\left(1+S_{B}{\hat{\Theta }}_{B}\right)-{\tilde{R}}_{E}\right)=\frac{\tilde{\bar{N}}\left({\hat{\Theta }}_{B}\right)}{{\hat{\Theta }}_{B}{\tilde{\bar{N}}}^{\prime }\left({\hat{\Theta }}_{B}\right)},$$

where the $S_{B}{\hat{\Theta }}_{B}$-terms and ${\hat{\Theta }}_{B}$-terms are separated into different sides of Equation (17). We define

(18) $$\begin{array}{ccc} & u & \overset{▵}{=}S_{B}{\hat{\Theta }}_{B}, \end{array}$$

(19) $$\begin{array}{cc} g(u) & \overset{▵}{=}\left(1+u\right)\left(\ln\left(1+u\right)-{\tilde{R}}_{E}\right)/u, \end{array}$$

(20) $$\begin{array}{cc} t({\hat{\Theta }}_{B}) & \overset{▵}{=}\tilde{\bar{N}}\left({\hat{\Theta }}_{B}\right)/{\hat{\Theta }}_{B}{\tilde{\bar{N}}}^{\prime }\left({\hat{\Theta }}_{B}\right). \end{array}$$

Then, Equation (17) is given by g(u)=t. From Equation (19), this relationship becomes $1+u=e^{t-\frac{t}{1+u}+{\tilde{R}}_{E}}$. Let $v=-\frac{t}{1+u}$, which is rewritten to $ve^{v}=-te^{-t-{\tilde{R}}_{E}}$, which is solved by $v=W_{0}\left(-te^{-t-{\tilde{R}}_{E}}\right)$ where $W_{0}\left(x\right)$ is the principal branch ($W_{0}\left(x\right)>-1$) of Lambert’s *W*-function. Thus, we have $1+S_{B}{\hat{\Theta }}_{B}=e^{t+W_{0}\left(-te^{-t-{\tilde{R}}_{E}}\right)-R_{E}}$.

Then, we solve the problem in Equation (14) by

(21) $$\begin{array}{ccc} & S_{B}\left({\hat{\Theta }}_{B}\right) & =\frac{e^{t+W_{0}\left(-te^{-t-{\tilde{R}}_{E}}\right)+{\tilde{R}}_{E}}-1}{{\hat{\Theta }}_{B}}, \end{array}$$

(22) $$\begin{array}{cc} {\hat{R}}_{B}\left({\hat{\Theta }}_{B}\right) & =t+W_{0}\left(-te^{-t-{\tilde{R}}_{E}}\right)+{\tilde{R}}_{E}, \end{array}$$

(23) $$\begin{array}{cc} \eta_{s}\left({\hat{\Theta }}_{B}\right) & =\frac{R_{B}\left({\hat{\Theta }}_{B}\right)-{\tilde{R}}_{E}}{\tilde{\bar{N}}\left({\hat{\Theta }}_{B}\right)}, \end{array}$$

where $t\left({\hat{\Theta }}_{B}\right)=\tilde{\bar{N}}\left({\hat{\Theta }}_{B}\right)/{\hat{\Theta }}_{B}{\tilde{\bar{N}}}^{\prime }\left({\hat{\Theta }}_{B}\right)$. We see that all equations are expressed only in terms of the parameter ${\hat{\Theta }}_{B}$. With the given $S_{B}$, the optimal ${\hat{R}}_{B}\left({\hat{\Theta }}_{B}\right)$ and $\eta_{s}\left({\hat{\Theta }}_{B}\right)$ can therefore be obtained.

As g(u), defined in Equation (19), can be expanded by $\ln\left(1+u\right)≃u-u^{2}/2+O\left(u^{3}\right)$, we give the low and high SNR asymptotes as follows:

**Remark** **1.** *As $S_{B}\to 0$ for finite M, the problem in Equation (14) is solved by*

(24) $$\begin{array}{ccc} & S_{B}\left({\hat{\Theta }}_{B}\right) & =\frac{t-1+\sqrt{{\left(t-1\right)}^{2}+2{\tilde{R}}_{E}}}{{\hat{\Theta }}_{B}}, \end{array}$$

(25) $$\begin{array}{cc} {\hat{R}}_{B}\left({\hat{\Theta }}_{B}\right) & =\ln\left(t+\sqrt{{\left(t-1\right)}^{2}+2{\tilde{R}}_{E}}\right), \end{array}$$

(26) $$\begin{array}{cc} \eta_{s}\left({\hat{\Theta }}_{B}\right) & =\frac{\ln\left(t+\sqrt{{\left(t-1\right)}^{2}+2{\tilde{R}}_{E}}\right)-{\tilde{R}}_{E}}{\tilde{\bar{N}}\left({\hat{\Theta }}_{B}\right)}, \end{array}$$

*where $t\left({\hat{\Theta }}_{B}\right)=\tilde{\bar{N}}\left({\hat{\Theta }}_{B}\right)/{\hat{\Theta }}_{B}{\tilde{\bar{N}}}^{\prime }\left({\hat{\Theta }}_{B}\right)$.*

**Proof.**  When $S_{B}\to 0$, we have $g\left(u\right)≃1+u/2-{\tilde{R}}_{E}/u$. By g(u)=t, $u=t-1+\sqrt{{\left(t-1\right)}^{2}+2{\tilde{R}}_{E}}$, our solutions then become Equations (24)–(26), as $u=S_{B}{\hat{\Theta }}_{B}$, $R_{B}=\ln\left(1+u\right)$, and $\eta_{s}=\left(R_{B}-{\tilde{R}}_{E}\right)/\tilde{\bar{N}}$. □

**Remark** **2.** *As $S_{B}\to \infty$ for finite M, the problem in Equation (14) is solved by*

(27) $$\begin{array}{ccc} & S_{B}\left({\hat{\Theta }}_{B}\right) & =\frac{e^{t+{\tilde{R}}_{E}}}{{\hat{\Theta }}_{B}}, \end{array}$$

(28) $$\begin{array}{cc} {\hat{R}}_{B}\left({\hat{\Theta }}_{B}\right) & =t, \end{array}$$

(29) $$\begin{array}{cc} \eta_{s}\left({\hat{\Theta }}_{B}\right) & =\frac{t-{\tilde{R}}_{E}}{\tilde{\bar{N}}\left({\hat{\Theta }}_{B}\right)}, \end{array}$$

*where $t\left({\hat{\Theta }}_{B}\right)=\tilde{\bar{N}}\left({\hat{\Theta }}_{B}\right)/{\hat{\Theta }}_{B}{\tilde{\bar{N}}}^{\prime }\left({\hat{\Theta }}_{B}\right)$.*

**Proof.**  When $S_{B}\to \infty$, g(u)≃ln(1+u). By g(u)=t, we have $u=e^{t+{\tilde{R}}_{E}}-1$. Then, Equations (27)–(29) can be achieved. □

High reliability is the fundamental requirement in a modern wireless communication system. Hence, we continue to consider the problem in Equation (14) with the following COP constraint:

(30) $$\begin{array}{c} \begin{array}{cc} \max_{R_{B}} & \;\eta_{s}\\ s.t. & \;P_{e}\leq P_{e}^{⋆}\\ & \;0\leq {\tilde{R}}_{E}\leq R_{B} \end{array}, \end{array}$$

where $P_{e}^{⋆}$ denotes the target COP. According to Equation (4), $P_{e}$ increases monotonically with increasing $R_{B}$. Hence, we know the COP constraint requires $R_{B}\leq R_{B}^{⋆}$, where

(31) $$R_{B}^{⋆}=\ln\left[1+\frac{S_{B}}{2}F_{\chi^{2}}^{-1}\left[P_{e}^{⋆},2K\right]\right],$$

in which $F_{\chi^{2}}^{-1}\left[\cdot \right]$ is the inverse function of the CDF of the chi-squared distribution.

As the solution of Equation (14) requires $d\eta_{s}/dR_{B}{|}_{{\hat{R}}_{B}}=0$ and $d^{2}\eta_{s}/dR_{B}^{2}{|}_{{\hat{R}}_{B}}<0$, (30) can be solved by:

(32) $$R_{B}^{†}=\min\left[{\hat{R}}_{B}\left({\hat{\Theta }}_{B}\right),R_{B}^{⋆}\right],$$

where the optimal point $R_{B}^{†}$ is always the maximum point in the feasible set of $R_{B}$.

### 4.2. Optimization of Secrecy Redundancy Rate

When the number of eavesdroppers is *M*, the maximum transmission number is *K* and code rate is given by ${\tilde{R}}_{B}$, we consider the problem of how to determine the secrecy redundancy rate which maximizes the EST:

(33) $$\begin{array}{c} \begin{array}{cc} \max_{R_{E}} & \;\eta_{s}\\ s.t. & \;0\leq R_{E}\leq {\tilde{R}}_{B} \end{array}, \end{array}$$

where $\eta_{s}$ is obtained by Equation (13). With the given ${\tilde{R}}_{B}$, the decoding threshold of main channel becomes ${\tilde{\Theta }}_{B}=e^{{\tilde{R}}_{B}-1}/S_{B}$. According to Equation (4),

(34) $${\tilde{P}}_{e}=\gamma_{r}\left(K,{\tilde{\Theta }}_{B}\right).$$

Thus, the EST of HARQ-CC becomes

(35) $$\eta_{s}=\frac{\left(1-{\tilde{P}}_{e}\right)}{\bar{N}}\cdot \left({\tilde{R}}_{B}-R_{E}\right)\cdot \left(1-P_{s}\right),$$

where ${\tilde{P}}_{e}$ and $\bar{N}$ are both determined, and $P_{s}$ is given in Equation (6) and approximated in Equation (12).

**Proposition** **1.** *$\eta_{s}$ is a log-concave function on $0\leq R_{E}\leq {\tilde{R}}_{B}$, with existing maximum value.*

**Proof.**  Take the natural logarithm of both sides of Equation (35),

(36) $$\ln\eta_{s}=\ln\left(1-{\tilde{P}}_{e}\right)-\ln\bar{N}+\ln\left({\tilde{R}}_{B}-R_{E}\right)+\ln\left(1-P_{s}\right),$$

where the first two parts in the RHS of Equation (36) are determined. In the third part, $\ln\left({\tilde{R}}_{B}-R_{E}\right)$ is a composition function $f=\ln\left(g\left(R_{E}\right)\right)$ on $0\leq R_{E}\leq {\tilde{R}}_{B}$, and $g\left(R_{E}\right)={\tilde{R}}_{B}-R_{E}$. $g\left(R_{E}\right)$ is obviously concave. Based on the convexity-preserving properties, $\ln\left({\tilde{R}}_{B}-R_{E}\right)$ is still concave on $0\leq R_{E}\leq {\tilde{R}}_{B}$. Finally, as $1-P_{s}$ is the $M^{\mathrm{th}}$ power of the CDF of a chi-squared distribution, which is logarithmic concave, $\ln\left(1-P_{s}\right)$ is concave. Therefore, $\eta_{s}$ is logarithmic concave with maximum value [31]. □

Therefore, the log-concave optimization problem given in Equation (33) can be converted to the following concave one:

(37) $$\begin{array}{c} \begin{array}{cc} \max_{R_{E}} & \;\ln\eta_{s}\\ s.t. & \;0\leq R_{E}\leq R_{B} \end{array}. \end{array}$$

Based on the above analysis, we know that if the optimal point ${\hat{R}}_{E}$ satisfies $d\ln\eta_{s}/dR_{E}{|}_{{\hat{R}}_{E}}=0$, then $\ln\eta_{s}$ and $\eta_{s}$ both have their maximum value at this value. From Equation (35),

(38) $$\begin{array}{c} \begin{array}{cc} \frac{d\eta_{s}}{dR_{E}} & =\frac{d}{dR_{E}}\left(\ln\left(1-{\tilde{P}}_{e}\right)-\ln\bar{N}+\ln\left({\tilde{R}}_{B}-R_{E}\right)+\ln\left(1-P_{s}\right)\right)\\ & =-\frac{1}{{\tilde{R}}_{B}-R_{E}}-\frac{1}{1-P_{s}}\cdot \frac{dP_{s}}{dR_{E}} \end{array}, \end{array}$$

where $P_{s}$ is approximated by Equation (12) and its first derivative is

(39) $$\frac{dP_{s}}{dR_{E}}≃-\frac{M\Theta_{E}^{\bar{N}-1}e^{R_{E}-\Theta_{E}}{\left(\gamma_{r}\left(\bar{N},\Theta_{E}\right)\right)}^{M-1}}{S_{E}\Gamma \left(\bar{N}\right)}.$$

Substituting Equations (12) and (39) into Equation (38) and letting $d\eta_{s}/dR_{E}=0$, we have the following fixed-point equation of the approximated ${\hat{R}}_{E}$:

(40) $${\hat{R}}_{E}≃{\tilde{R}}_{B}-\frac{S_{E}\gamma \left(\bar{N},{\hat{\Theta }}_{E}\left({\hat{R}}_{E}\right)\right)e^{{\hat{\Theta }}_{E}\left({\hat{R}}_{E}\right)}}{Me^{{\hat{R}}_{E}}{\left({\hat{\Theta }}_{E}\left({\hat{R}}_{E}\right)\right)}^{\bar{N}-1}},$$

where ${\hat{\Theta }}_{E}\left({\hat{R}}_{E}\right)=\left(e^{{\hat{R}}_{E}}-1\right)/S_{E}$. Some classical techniques, such as the fixed-point iterative method, are suitable for solving the above equation.

**Remark** **3.** *As $S_{E}\to 0$, we obtain ${\hat{R}}_{E}=0$.*

**Proof.**  Since γ(s,x)→Γ(s) if x→∞, when $S_{E}\to 0$, we have $\gamma_{r}\left(\bar{N},\Theta_{E}\right)\to 1$. Hence, from Equation (12), $P_{s}\to 0$ and Equation (35) become

(41) $$\eta_{s}=\frac{1-{\tilde{P}}_{e}}{\bar{N}}\cdot \left({\tilde{R}}_{B}-R_{E}\right).$$

It is easy to find that the maximum value of $\eta_{s}$, $\frac{\left(1-{\tilde{P}}_{e}\right)\cdot {\tilde{R}}_{B}}{\bar{N}}$, is obtained when $R_{E}=0$. □

**Remark** **4.** *As $S_{E}\to \infty$, ${\hat{R}}_{E}$ can be obtained by solving the fixed-point equation,*

(42) $${\hat{R}}_{E}={\tilde{R}}_{B}-\frac{e^{{\hat{R}}_{E}}-1}{M\cdot e^{{\hat{R}}_{E}}\cdot \bar{N}}.$$

**Proof.**  If $S_{E}\to \infty$, ${\hat{\Theta }}_{E}\to 0$. Applying $\frac{\gamma (s,x)}{x^{s}}\to \frac{1}{s}$ when x→0, we have

(43) $$\lim_{{\hat{\Theta }}_{E}\to 0}\frac{\gamma \left(\bar{N},\Theta_{E}\right)}{{\left({\hat{\Theta }}_{E}\right)}^{\bar{N}}}=\frac{1}{\bar{N}}.$$

Substituting Equation (43) into Equation (40), Equation (42) can be obtained. □

When the SOP constraint is required (e.g., in some special application scenarios), the optimization problem of secrecy redundancy rate aiming to enhance the EST becomes:

(44) $$\begin{array}{c} \begin{array}{cc} \max_{R_{E}} & \;\eta_{s}\\ s.t. & \;P_{s}\leq P_{s}^{⋆}\\ & \;0\leq R_{E}\leq {\tilde{R}}_{B} \end{array}, \end{array}$$

where $P_{s}^{⋆}$ denotes the target SOP. According to Equation (12), $P_{s}$ decreases monotonically with increasing $R_{E}$. Hence, we know the SOP constraint requires $R_{E}\geq R_{E}^{⋆}$, and

(45) $$R_{E}^{⋆}=\ln\left[1+\frac{S_{E}}{2}F_{\chi^{2}}^{-1}\left[{\left(1-P_{s}^{⋆}\right)}^{\frac{1}{M}},2K\right]\right],$$

where $F_{\chi^{2}}^{-1}\left[\cdot \right]$ is the same inverse function of the CDF of the chi-squared distribution as in Equation (31).

Since EST in Equation (35) has been proven to be log-concave on $R_{E}$ and $d\ln\eta_{s}/dR_{E}{|}_{{\hat{R}}_{E}}=0$, Equation (44) can be solved by

(46) $$R_{E}^{†}=\max\left[{\hat{R}}_{E},R_{E}^{⋆}\right],$$

where the optimal point $R_{E}^{†}$ is always the maximum point in the feasible set of $R_{E}$.

### 4.3. Optimization of the Rate Pair $\left(R_{B},R_{E}\right)$

In this part, we discuss a more general problem, which optimizes both the code rate and secrecy redundancy rate—that is, the rate pair ($R_{B},R_{E}$)—with the EST criteria. When multiple eavesdroppers and limited retransmission number are still considered, this optimization problem is given by

(47) $$\begin{array}{c} \begin{array}{cc} \max_{R_{B},R_{E}} & \;\eta_{s}\\ s.t. & \;0\leq R_{E}\leq R_{B} \end{array}, \end{array}$$

where $\eta_{s}$ is obtained by Equation (13), $P_{e}$ and $P_{s}$ are given by Equations (4) and (12), respectively. As the expression of $\eta_{s}$ is extremely complicated and its concavity is difficult to prove, we proposed an iterative algorithm to determine the rate pair ($R_{B}$, $R_{E}$).

In brief, the optimization problem in Equation (47) can be tackled by iteratively adapting $R_{B}$ and $R_{E}$ separately until the EST gain denoted by δ is no greater than ϵ, where ϵ is a preassigned small positive real number (e.g., $10^{-3}$). Specifically, it is first assumed that $\delta =\eta_{s}^{\left(1\right)}-\eta_{s}^{\left(0\right)}>\epsilon$, where $\eta_{s}^{\left(0\right)}$ and $\eta_{s}^{\left(1\right)}$ denote the optimal EST before and after each iteration, respectively. Here, we initialize them as $\eta_{s}^{\left(0\right)}=0$ and δ=1. The optimal rates are initialized as ${\hat{R}}_{B}=0$ and ${\hat{R}}_{E}=0$. Next, using ${\tilde{R}}_{E}={\hat{R}}_{E}$, we solve the optimization of $R_{B}$ in Equation (14), while the optimal point ${\hat{R}}_{B}$ is obtained by Equations (21)–(23). Then, using ${\tilde{R}}_{B}={\hat{R}}_{B}$, we solve the optimization of $R_{E}$ in Equation (33), while the optimal point ${\hat{R}}_{E}$ is obtained by Equation (40). After this iteration, we computed the maximum EST, $\eta_{s}\left({\hat{R}}_{B},{\hat{R}}_{E}\right)$, by Equation (13) and set $\eta_{s}^{\left(1\right)}=\eta_{s}\left({\hat{R}}_{B},{\hat{R}}_{E}\right)$ to evaluate the EST gain by $\delta =\eta_{s}^{\left(1\right)}-\eta_{s}^{\left(0\right)}$. Simultaneously, $\eta_{s}^{\left(0\right)}$ is updated by $\eta_{s}^{\left(1\right)}$ for next iteration. The iterations continue if δ>ϵ; otherwise, the optimal rate pair (${\hat{R}}_{B},{\hat{R}}_{E}$) is output. This algorithm giving the ϵ-suboptimal solution is summarized in Algorithm 1.
**Algorithm 1** Iterative optimization of ($R_{B},R_{E}$) for solving Equation (47).**Input:**$\epsilon =10^{-3}$, ${\hat{R}}_{B}=0$, ${\hat{R}}_{E}=0$, $\eta_{s}^{\left(0\right)}=0$, δ=1;1:**while**δ>ϵ**do**2: ${\tilde{R}}_{E}⇐{\hat{R}}_{E}$;3: Compute ${\hat{R}}_{B}$ by Equations (21)–(23)4: ${\tilde{R}}_{B}⇐{\hat{R}}_{B}$
5: Compute ${\hat{R}}_{E}$ by Equation (40)6: Compute $\eta_{s}\left({\hat{R}}_{B},{\hat{R}}_{E}\right)$ by Equation (13)7: $\eta_{s}^{\left(1\right)}⇐\eta_{s}\left({\hat{R}}_{B},{\hat{R}}_{E}\right)$
8: $\delta ⇐\eta_{s}^{\left(1\right)}-\eta_{s}^{\left(0\right)}$
9: $\eta_{s}^{\left(0\right)}⇐\eta_{s}^{\left(1\right)}$
10:**end while****Output:** (${\hat{R}}_{B},{\hat{R}}_{E}$);

Then, we reconsider Equation (47) when COP and SOP constraints are both required. The optimization becomes:

(48) $$\begin{array}{c} \begin{array}{cc} \max_{R_{B},R_{E}} & \;\eta_{s}\\ s.t. & \;P_{e}\leq P_{e}^{⋆}\\ & \;P_{s}\leq P_{s}^{⋆}\\ & \;0\leq R_{E}\leq R_{B} \end{array}, \end{array}$$

where $P_{e}^{⋆}$ and $P_{s}^{⋆}$ denote the target COP and SOP, respectively. This problem can be solved by a modified version of Algorithm 1, in which ${\hat{R}}_{B}$ and ${\hat{R}}_{E}$ are replaced by $R_{B}^{†}$ and $R_{E}^{†}$, computing Equations (32) and (46). In other words, the optimal rate pair should be selected among its feasible set.

## 5. Numerical Results

In this section, a wireless HARQ-CC system with Alice, Bob and multiple eavesdroppers, Eve1, *…*, EveM, were considered, as shown in Figure 1. Under this system model, some typical results were demonstrated to evaluate the security performance. These related performance metrics include SOP, EST and optimal rate, with a given number of eavesdropper *M* and maximum transmission number *K*.

### 5.1. SOP Results

**Figure 2:**
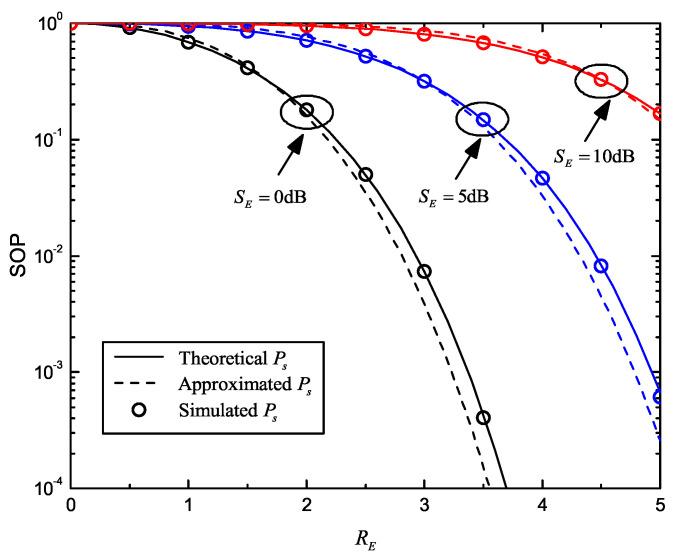
Secrecy outage probability (SOP) versus $R_{E}$ for different average received signal-to-noise ratio (SNR) of wiretap channel with multiple eavesdroppers; $S_{E}\in \left\{0\mathrm{dB},5\mathrm{dB},10\mathrm{dB}\right\}$, $S_{B}=20\mathrm{dB}$, $R_{B}=5$, K=4 and M=2.

In Figure 2, we plot the SOP curves versus $R_{E}$ for different $S_{E}$, which are determined by both the main and wiretap channels. The parameters were set as $S_{B}=20\;\mathrm{dB}$, $R_{B}=5$, K=4, M=2 and $S_{E}=0\;\mathrm{dB},\;5\;\mathrm{dB},\;10\;\mathrm{dB}$. Theoretical and approximated $P_{s}$ were obtained by Equations (6) and (12), respectively. First, we found that the simulation curves precisely match those of theoretical $P_{s}$, while their differences from the approximated $P_{s}$ were limited. Then, SOP monotonically decreases with increasing $R_{E}$, which means that security will be enhanced by more secrecy redundancy. Furthermore, it should be pointed out that, in order to maintain the same SOP value, a larger $R_{E}$ is required when $S_{E}$ increases. In other words, although the wiretap channel is better, we need more secrecy redundancy to ensure the same level of security.

**Figure 3:**
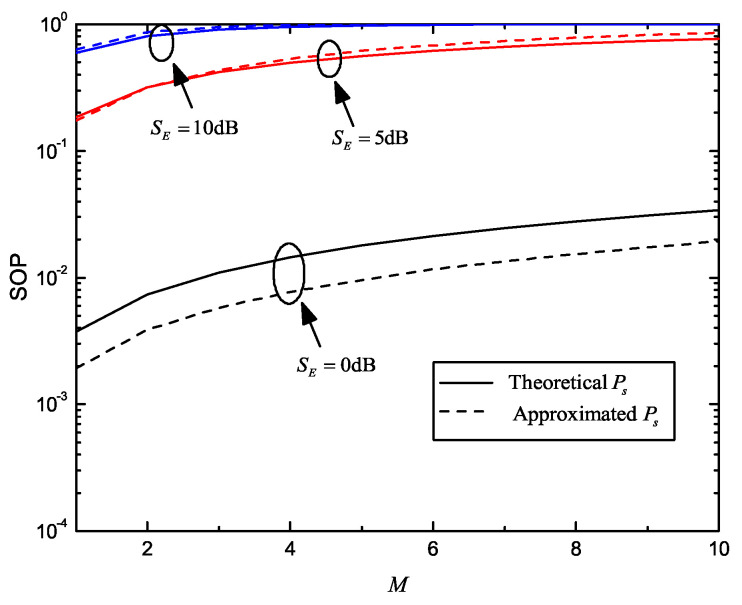
SOP versus *M* for different average received SNR of wiretap channel with multiple eavesdroppers; $S_{E}\in \left\{0\;\mathrm{dB},5\;\mathrm{dB},10\;\mathrm{dB}\right\}$, $S_{B}=20\;\mathrm{dB}$, $R_{B}=5$, $R_{E}=3$ and K=4.

Figure 3 shows the SOP versus *M* for different $S_{E}$. For all curves, $S_{B}=20\;\mathrm{dB}$, $R_{B}=5$, $R_{E}=3$, K=4 and $S_{E}=0\;\mathrm{dB},5\;\mathrm{dB},10\;\mathrm{dB}$. Theoretical and approximated $P_{s}$ are also obtained by Equations (6) and (12); their differences are also limited, considering the use of logarithmic co-ordinate. SOP slowly rises with increasing *M*, which means that secrecy performance worsens when more eavesdroppers exist. On the other hand, when $S_{E}$ increases, SOP with fixed *M* increases sharply. This means the condition of wiretap channel has more influence on the security.

### 5.2. Optimization Results of Code Rate

**Figure 4:**
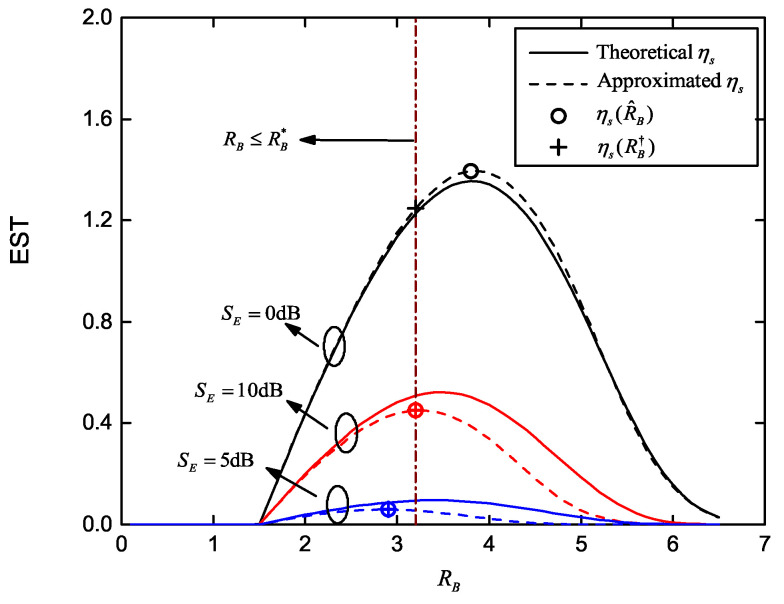
Effective secrecy throughput (EST) versus $R_{B}$ for different average received SNR of wiretap channel and multiple eavesdroppers, with and without the COP constraint; $S_{E}\in \left\{0\;\mathrm{dB},5\;\mathrm{dB},10\;\mathrm{dB}\right\}$, $P_{e}^{⋆}=10^{-4}$, $S_{B}=20\;\mathrm{dB}$, ${\tilde{R}}_{E}=1.5$, M=2 and K=4.

Figure 4 shows the EST curves versus $R_{B}$ for different $S_{E}$ where the maximum ESTs are marked with and without the COP constraint. Parameters are set as $S_{B}=20\;\mathrm{dB}$, ${\tilde{R}}_{E}=1.5$, M=2, K=4 and $S_{E}=0\;\mathrm{dB},\;5\;\mathrm{dB},\;10\;\mathrm{dB}$. Target COP is $P_{e}^{⋆}=10^{-4}$ when it is considered. Theoretical and approximated $\eta_{s}$ curves are generated according to $P_{s}$ and the approximated $P_{s}$, respectively. It can be observed that the difference between the theoretical and approximated $\eta_{s}$ is limited, especially the maximum value. EST curves increase monotonically to the maximum point with increasing $R_{B}$, and then decrease monotonically. Hence, their slopes are positive when $R_{B}$ is less than its optimal value ${\hat{R}}_{B}$, and negative when $R_{B}>{\hat{R}}_{B}$. The maximum $\eta_{s}\left({\hat{R}}_{B}\right)$, using the parameterized close-form solution in Equations (21)–(23), are plotted in Figure 4. These results well match the maximum $\eta_{s}$ and maximum approximated $\eta_{s}$ without COP constraint. Considering the COP constraint $P_{e}<P_{e}^{⋆}$, we state the feasible set $R_{B}\leq R_{B}^{⋆}$. The corresponding maximum EST values, $\eta_{s}\left(R_{B}^{†}\right)$, are also plotted, in which $R_{B}^{†}$ equals the minimum of ${\hat{R}}_{B}$ and $R_{B}^{⋆}$.

**Figure 5:**
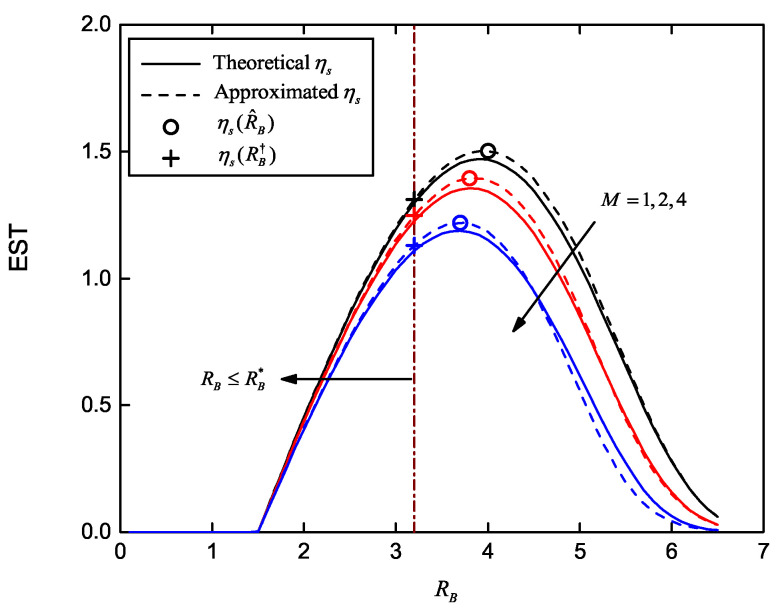
EST versus $R_{B}$ for different number of eavesdroppers, with and without the COP constraint; $M\in \left\{1,2,4\right\}$, $S_{B}=20\;\mathrm{dB}$, $S_{E}=0\;\mathrm{dB}$, $P_{e}^{⋆}=10^{-4}$, ${\tilde{R}}_{E}=1.5$ and K=4.

In Figure 5, we plot the EST versus $R_{B}$ for different *M*. For all curves, $S_{B}=20\;\mathrm{dB}$, $S_{E}=0\;\mathrm{dB}$, ${\tilde{R}}_{E}=1.5$ and K=4. The target COP is still $P_{e}^{⋆}=10^{-4}$. Theoretical and approximated $\eta_{s}$ also match well. $\eta_{s}\left({\hat{R}}_{B}\right)$ and $\eta_{s}\left(R_{B}^{†}\right)$ illustrate maximum ESTs without and with the COP constraint, respectively; the differences between their maximum and optimized values are all limited. Then, it is critical to point out that, all these $\eta_{s}$ decrease obviously with increasing *M*. Similarly to SOP, more eavesdroppers worsen secrecy performance, including the EST.

### 5.3. Optimization Results of Secrecy Redundancy Rate

**Figure 6:**
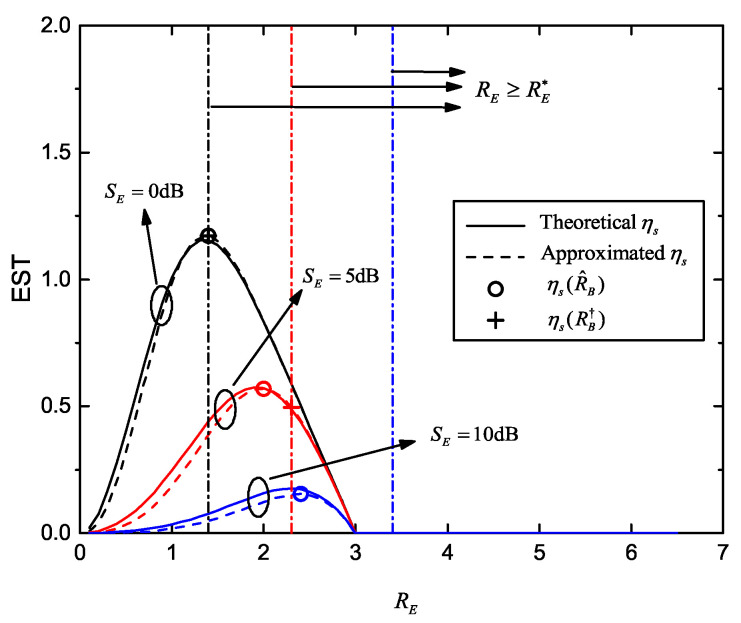
EST versus $R_{E}$ for different average received SNR of wiretap channel and multiple eavesdroppers, with and without the SOP constraint; $S_{E}\in \left\{0\;\mathrm{dB},5\;\mathrm{dB},10\;\mathrm{dB}\right\}$, $P_{s}^{⋆}=10^{-1}$, $S_{B}=20\;\mathrm{dB}$, ${\tilde{R}}_{B}=3$, K=4 and M=2.

In Figure 6, we plot the EST curves versus $R_{E}$, as well as maximum ESTs corresponding to calculated optimal $R_{E}$ with and without the SOP constraint. The channel conditions are $S_{B}=20\;\mathrm{dB}$, $S_{E}=0\;\mathrm{dB},5\;\mathrm{dB}$ and 10dB. The SOP constraint is $P_{s}^{⋆}\leq 10^{-1}$, when considered. The other parameters are set as ${\tilde{R}}_{B}=3$, K=4 and M=2. Compared with the theoretical $\eta_{s}$, we verified that our approximated $\eta_{s}$ is relatively accurate. The maximum ESTs without SOP constraint (i.e., $\eta_{s}\left({\hat{R}}_{E}\right)$), are obtained by the fixed-point method in Equation (40). They precisely match the maximum value of the approximated $\eta_{s}$ curves. When $P_{s}^{⋆}$ is involved, we also state the feasible set, $R_{E}\geq R_{E}^{⋆}$. The solutions $\eta_{s}\left(R_{E}^{†}\right)$ computed by Equation (46) are plotted. It is worth noting that, $\eta_{s}\left(R_{E}^{†}\right)=\eta_{s}\left({\hat{R}}_{E}\right)=\eta_{s}\left(R_{E}^{⋆}\right)$ for $S_{E}=0\;\mathrm{dB}$, $\eta_{s}\left(R_{E}^{†}\right)=\eta_{s}\left({\hat{R}}_{E}\right)$ for $S_{E}=5\;\mathrm{dB}$, and no feasible solution arrives for $S_{E}=10\;\mathrm{dB}$, under the given $P_{s}^{⋆}$.

**Figure 7:**
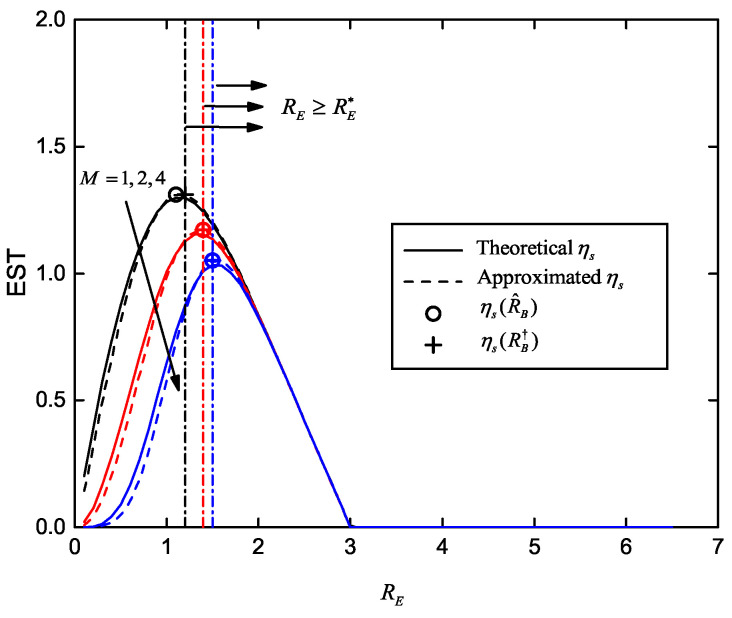
EST versus $R_{E}$ for different number of eavesdroppers, with and without the SOP constraint; $M\in \left\{1,2,4\right\}$, $S_{B}=20\;\mathrm{dB}$, $S_{E}=0\;\mathrm{dB}$, $P_{s}^{⋆}=10^{-1}$, ${\tilde{R}}_{B}=3$ and K=4.

Figure 7 shows the EST versus $R_{E}$ for different number of eavesdroppers with and without the SOP constraint, and corresponding optimized ESTs. The channel conditions and maximum transmission number are same as the parameters in Figure 6. The SOP constraint is $P_{s}^{⋆}=10^{-1}$. The three groups of EST curves are obtained with M=1,2,4, respectively. With an increasing *M*, we found that the optimal $R_{E}$ rises and maximum EST reduces, which indicates that in order to meet the SOP requirement, we need an increased secrecy redundancy when more eavesdroppers exist; thus EST decreases.

### 5.4. Optimization Results of the Rate Pair ($R_{B}$, $R_{E}$)

**Figure 8:**
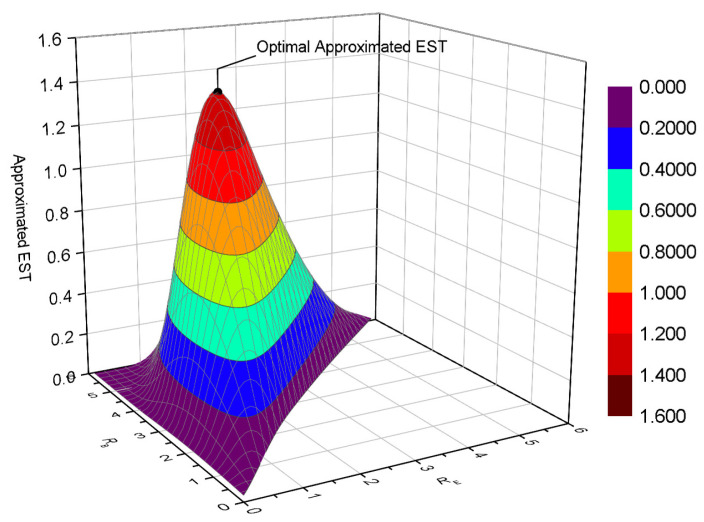
EST versus ($R_{B}$, $R_{E}$) for multiple eavesdroppers, without COP and SOP constraint; $S_{B}=20\;\mathrm{dB}$, $S_{E}=0\;\mathrm{dB}$, M=2, and K=4.

Figure 8 depicts the EST versus $R_{B}$ and $R_{E}$ for multiple eavesdroppers, without COP and SOP constraint. For simplicity, only the approximated EST is plotted here, while its accuracy was verified by Figure 4, Figure 5, Figure 6 and Figure 7. The maximum EST is also marked, which was obtained by Algorithm 1. We observed that the optimization is solved precisely, which confirms the effectiveness of Algorithm 1. In our simulation, we also found that the iteration number is small (only about four iterations were needed).

**Figure 9:**
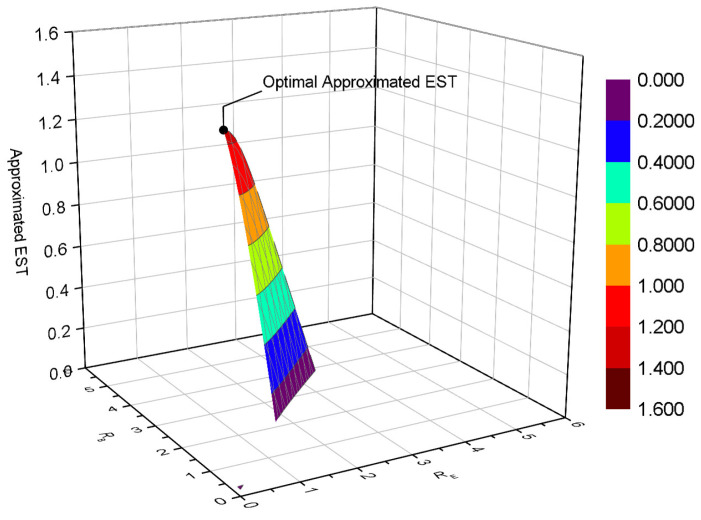
EST versus ($R_{B}$, $R_{E}$) for multiple eavesdroppers, with COP and SOP constraints; $S_{B}=20\;\mathrm{dB}$, $S_{E}=0\;\mathrm{dB}$, $P_{e}^{⋆}=10^{-4}$, $P_{s}^{⋆}=10^{-1}$, M=2, and K=4.

Finally, we give the surface of the EST versus $R_{B}$ and $R_{E}$ with COP and SOP constraints in Figure 9. The same parameters as Figure 8 are configured here, except for $P_{e}^{⋆}=10^{-4}$ and $P_{s}^{⋆}=10^{-1}$. The optimal EST is located at the maximum value of the approximated EST, which proves that our solution for Equation (48) works well. COP and SOP constraints, in fact, define a 2-dimensional feasible space for rate adaption.

## 6. Conclusions

In this paper, we discussed the rate adaption of secure transmissions in HARQ-CC system, with multiple eavesdroppers and limited latency. We first presented some critical secrecy performance metrics, including COP, SOP and EST. Then, three optimization problems were derived using a parameterized closed-form solution, a fixed-point method and an iterative algorithm, respectively. Finally, numerical and simulated results demonstrated that our proposed methods improved secrecy performance efficiently by optimizing code rate, secrecy redundancy rate and both of them paired. We also concluded that more eavesdroppers worsen the secrecy performance, but channel condition plays a more significant role.
